# In-vitro assessment of antioxidant and antimicrobial activities of methanol extracts and essential oil of *Thymus hirtus sp. algeriensis*

**DOI:** 10.1186/1476-511X-13-114

**Published:** 2014-07-14

**Authors:** Guesmi Fatma, Ben Farhat Mouna, Mejri Mondher, Landoulsi Ahmed

**Affiliations:** 1 Laboratory of Biochemical and Molecular Biology, Faculty of Science of Bizerte, University of Carthage, Bizerte, Tunisia; 2 Higher Institute of Technological Studies (ISET), Mogran, Zaghouan, Tunisia

**Keywords:** Radical scavenging effect, Phenolic compounds, Essential oil, Antioxidant activity, Antibacterial activity

## Abstract

**Background:**

Owing to the complexity of the antioxidant materials and their mechanism of actions, it is obvious that no single testing method is capable of providing a comprehensive picture of the antioxidant profile. The essential oil of the *Thymus* specie may still possess other important activities in traditional medicine, it can be used in the treatment of fever and cough. This essential oil may also have an anticancer activity.

**Methods:**

The essential oils aerial parts hydrodistilled from *Thymus hirtus sp. algeriensis*, were characterised by GC/MS analysis and the methanolic extracts were chemically characterized by HPLC method. The essence of thyme was evaluated for its antioxidant and antibacterial activity.

**Result:**

The Terpinen-4-ol are the principal class of metabolites (33.34%) among which 1.8-cineole (19.96%) and camphor (19.20%) predominate. In this study, quantitative values of antioxidant activity of crude methanolic extracts of *Thymus hirtus sp. algeriensis* were investigated. The essential oils was screened for their antibacterial activity against six common pathogenic microorganisms (*Escherichia coli*, *Pseudomonas aeruginosa*, *Salmonella enteridis*, *Staphylococcus aureus*, *Bacillus subtilis* and *Listeria monocytogenes*) by well diffusion method and agar dilution method (MIC). All the essences were found to inhibit the growth of both gram (+) and gram (−) bacteria organisms tested. These activities were correlated with the presence of phenolic compounds in active fractions. HPLC confirmed presence of phenolic compounds in methanol extracts.

**Conclusion:**

Methanol extracts and essential oils from aerial parts of *Thymus hirtus sp. algeriensis*, were examined for their potential as antioxidants. The technique for measuring antioxidant activity, which was developed using DPPH, ABTS and β-carotene bleaching, produced results as found in established literatures. The present results indicate clearly that methanol extracts and essential oils from *Thymus hirtus sp. algeriensis* possess antioxidant properties and could serve as free radical inhibitors or scavengers, acting possibly as primary antioxidants, also their essential oil have an antibacterial effect.

## Background

*Thymus hirtus sp. algeriensis* (thyme), locally known as “Mougecha” or “Mazoukcha”, a member of the Lamiaceae family, is the most widespread North African species. In Tunisia, *Thymus hirtus sp. algeriensis* populations are distributed from the sub-humid to the lower arid bioclimates and grow at altitudes ranging from 120 to 1100 [1]. The species grow on poor fertile calcareous soils and occurs in scattered and small populations. *Thymus hirtus sp. algeriensis* is a short lived shrub, described as diploid (2n = 2 × = 30) and gynodioecious species [2]. It is often pollinated by bees (allogamous species); however self-pollination may occur in hermaphrodites [3]. The genus *Thymus* has numerous species and varieties and their essential oil composition have been studied earlier [4]; consists of about 350 species of perennial, aromatic herb and sub shrubs native to Europe and North Africa. Various species of thyme is used all over the globe as condiments, ornamentals and sources of essential oil [5]. *Thymus hirtus sp. algeriensis* is largely used, fresh or dried, only as a culinary herb and widely used in Tunisian folk medicine as anti-inflammatory, anti-diarrheic and anti-bronchic agents. Furthermore, this plant is widely used in folk medicine against illnesses of the digestive tube and antiabortion [6]. Essential oil of this specie was found to possess an interesting inhibitory activity towards angiotensin I-converting enzyme suggesting the potential of this plant as an antihypertensive agent [7].

Thyme oil is among the world’s top ten essential oil, displaying antimicrobial [8], antimycotic, antioxidative [7], food preservative, antifungal [9] and mammalian age-delaying properties [10]. A relatively high level of differentiation (F_ST_ = 0.146) between some population of *Thymus hirtus sp. algeriensis* associated with a restricted gene flow among populations (N_m_ = 2.385) was revealed according to the geographical regions [3] and to the vegetative stage [11]. Due to application of *Thymus hirtus sp. algeriensis* growing wild in Tunisia in folk medicine, the purpose of the present work was to evaluate the antioxidant and the antibacterial potential of their essential oils and methanolic extracts against an epidemiological relevant group of bacterial food-borne pathogens and relate it with their chemical composition, for further application in food and pharmaceutical industries as natural valuable products. All extracts were chemically characterized by HPLC method in order to find the connection between their activity and chemical composition of extracts.

## Material and methods

### Plant material

The flowering aerial parts of the studied Thymus species were collected randomly from three different locations in Tunisia, in March 2010. Gafsa (MG) (in the high lands); Tamerza (MT) (in the high lands) and Kairouan (MOK) (near to dam) (Table 1). The taxonomic identification of plant species were confirmed by the forest engineer of Bouhedma Natural Park, and a voucher specimen was deposited at the herbarium of the Laboratory of Medicinal Plants (INAT). The dried aerial parts were powdered and then used for extraction.

**Table 1:** **Collection sites of cultivated *Thymus hirtus sp.algeriensis *** **and their eco-geographical characteristics**

**No.**	**Collection site**	**Code**	**Bioclimatic stage**	**Soil pH**	**Rainfall (mm/year)**	**Temperature (°C/year)**	**Geographical location**		
							**Longitude (N)**	**Latitude (E)**	**Altitude (m)**
1	Gafsa	MG	Semi-arid	8.1	220-400	11-51.8	9°05’00”	34°46’67”	340
2	Tamerza	MT	Arid	7.5	200	12-50	7°95’00”	36°38’85”	441
3	Kairouan	MOK	Semi-arid	1-14	400-800	19-35	36°33’84”	10°51’52”	57.6

### Preparation of the extracts

Briefly, 9 g of powdered aerial parts were continuously extracted in absolute methanol for 8 h using a Soxhlet apparatus. The extracts were concentrated to dryness using a rotary evaporator. The extract yields (w/w) were 24.2% for MT, 15% for MOK and 18% for MG.

### Isolation of the essential oil

A portion (100 g) of dried and ground aerial parts of *Thymus hirtus sp. algeriensis* were submitted to water-distillation for 3 h using a Clevenger-type apparatus. Sample was dried over anhydrous Na_2_SO_4_ and kept in a refrigerator (4°C) for subsequent experiments. The thyme essential oils (yields of MT, MG and MOK are respectively 10.82% (v/w), 2.36% (v/w) and 5.75% (v/w)); was analyzed using a fused silica capillary column, HP5-MS.

### GC/MS analysis conditions

Samples of 1 μl (dilution in hexane 10%) were subjected to analysis by GC-MS. The GC analysis was performed on a model 7890 A (series II) gas chromatograph, with a flame ionisation detector (FID) and split ratio of 1:50 using a fused silica capillary column, HP5-MS (30 m × 250 μm i.d., 0.25 μm film thickness). The injector or detector temperature for each analysis was about 250°C, plus the carrier gas was helium at a flow rate of 0.8 ml/min. The peak areas were measured by electronic integration, and the relative amounts of the individual components based on the peak areas. The GC-MS was carried out on an Agilent model 5975 C mass spectrometry operating in the ionizing energy mode at 70 eV, combined with the GC described above.

The temperature of the column was programmed from 60°C to 240°C at 4°C/min. The injector and ion source temperatures were the same as mentioned above. Scanning from m/z 50–550 at 2.91 scan/s. The quadrupole temperature was 150°C, and the electron multiplier voltage was maintained at 1188 V. The constituents were identified by comparison of their retention indices with those of the literature (Adams, 1995). The retention indices were determined in relation to a homologous series of *n*-alkanes (C_10_–C_15_) under the same operating conditions.

### High performance liquid chromatographic identification and quantification of phenolic and flavonoid compounds in plant extracts

The HPLC was performed using KNAUER chromatographic system; equipped with a diode array UV detector model 2500. Eurospher 100–5 C_18_ column (250 × 4.6 mm with percolumn). The mobile phase was methanol; acetonitrile (50:50 v/v) (solvent A) and a mixture of water; acetic acid (97:3 v/v) (solvent B) at a flow rate of 1 ml/min. The solvent gradient changed according to the following conditions: from 5% to 30% A for 25 min; from 30% A to 38% A for 10 min; from 38% A to 45% A for 10 min; from 45% A to 53% A for 5 min; finally, isocratic for 60% A to 65% A for 5 min. Eluates were detected at 280 nm [12].

Phenolic compounds quantification was achieved by measuring the absorbance at 280 nm recorded the chromatograms relative to external standards. Phenolic compounds content were expressed in microgram per gram of dry plant material weight.

Total phenolic was determined by a miniaturisation of method developed by Singleton [13]. Appropriately diluted (15 fold), thyme extracts (50 μl) was mixed with 400 μl of Folin-Ciocalteu reagent (10%) and 50 μl distilled water and allowed to stand at room temperature for 5 min. After 3 to 8 min, soduim bicarbonate solution (7, 5%, 500 μl) was added to the mixture. Afterwards, the blue mixture was incubated at room temperature for 1 h. Absorption at 725 nm was measured using a spectrophotometer. The total phenolic content was expressed as gallic acid equivalents (GAE). The concentrations of phenolic compounds were calculated according to the following equation that was obtained from the standard gallic acid graph:

$$y\;=\;0.0016x\;\left(R^{2}\;=\;0.\;8675\right).$$

The determination of flavonoids was performed by Dewanto [14]. To 250 μl of diluted thyme extract, 5% sodium nitrite solution (75 μl) was added. Tubes were incubated at ambient temperature for 6 min, and then 10% of aluminium chloride solution (150 μl) was added to the mixture, followed by 500 μl of 1 M sodium hydroxide. Immediately, the volume of reaction mixture was made to 2, 5 ml with distilled water. The mixture was thoroughly vortexed and the absorbance of the pink colour developed was determined at 510 nm. A calibration curve was prepared with rutin and the results were expressed as rutin equivalents (RE).

The concentrations of flavonoids were calculated according to the following equation that was obtained from the standard rutin graph.

### Antioxidant activity

#### DPPH radical-scavenging activity

DPPH is a stable nitrogen radical that bears no similarity to the highly reactive and transient peroxyl radicals involved in lipid peroxidation. Antioxidant assay is based on measurement of the loss of DPPH color after reaction with test compounds [15], and the reaction is monitored by a spectrometer.

To methanolic solution (50 μl) of antioxidants (or essential oils) we added methanolic solution of DPPH (2 ml of 6.10^−5^ M). The antioxidant tests were based on the measurement of the loss of DPPH colour at 515 nm. The methanol is used to make at zero the spectrophotometer. BHT was used for comparative purposes.

All determinations were performed in triplex.

Free radical scavenging capacity was calculated by the following equation:

$$\%\;\mathrm{Inhibition}\;=\;\left(A_{\mathrm{blank}}–\;A_{\mathrm{sample}}/A_{\mathrm{blank}}\right)\;\times \;100$$

Where Ablank and Asample stand for absorption of the blank sample and absorption of tested extract solution respectively.

Extract concentration that provide 50% inhibition (IC_50_) and expressed in μg extract/ml was calculated from the graph plotted inhibition percentages against tested samples extracts.

The higher the IC_50_, the lower is the antioxidant activity of the examined sample.

#### 2, 2 -Azinobis-3-ethylbenzothiazoline-6-sulfonate (ABTS) radical scavenging assay

The total antioxidant activity of extracts was measured using the ABTS assay, as described by Miller [16] to test biological samples and then was widely applied to test food and natural water-soluble phenolics. The idea of this method is to monitor the decay of the radical cation ABTS^.+^ produced by the oxidation of 2,2^′^-azinobis (3-ethylbenzothiaziline-6-sulfonate) (ABTS) caused by the addition of a phenolic-containing sample. ABTS^. +^ has a strong absorption in the range of 600–750 nm that can be easily determined spectrophotometrically. In the absence of phenolics, ABTS^. +^ is rather stable, but it reacts energetically with a H-atom donor, such as phenolics, being converted into a non-colored form of ABTS. The authors determined the quantity of ABTS^. +^ consumed due to reaction with phenolic-containing sample.

ABTS^. +^ reacts with any hydroxylated aromatics independently of their real antioxidative potential. The ABTS value was based on the ability of the antioxidant to scavenge the blue-green 2,2^′^-azinobis 3-ethylbenzothiazoline-6-sulfonate (ABTS^.+^) radical cation relative to the ABTS^.+^ scavenging ability of BHT. ABTS^. +^ L’ABTS¨^+^ is generated after chemical oxydation reaction with potassium persulfate (K_2_S_2_O_8_) (2. 45 mM).

The concentration of radical solution ABTS blue-green is adjusted with methanol to an absorbance of 0.700 ± 0.020 (mean ± SD) at 734 nm. To 280 μl of this solution of ABTS¨^+^ we added 20 μl of extracts (or essential oils) or BHT or solvent. BHT was used for comparative purposes. The mixture is incubated during 5 min à 30°C, and absorbance is measured at 734 nm. All determinations were performed in triplicate.

#### β-carotene/linoleic acid assay

β-carotene is another important fat soluble antioxidant that quenches sites localised within the hydrophobic region of biological membranes, contrasting with the scavenging activity of α-tocopherol close to the membrane surface [17].

The antioxidant capacity was estimated by thermally induced β-carotene bleaching assay, as described by Pratt [18] with some modifications. The assay reagent was prepared by mixing 0.5 mg of β-carotene with 1 ml of chloroform, 25 μl of linoleic acid and 200 mg of Tween 40 (polyoxyethylenesorbitanmonopalmitate). Chloroform was removed at 50°C, under vacuum, using a rotary evaporator (Heidolph, Germany). The resulting mixture was immediately diluted with 100 ml of oxygenated distilled water. Aliquots (2500 μl) of this emulsion were transferred into different test tubes containing 350 μl of test samples. The tubes were shaken and incubated at 50°C in a water bath.

BHT was used for comparative purposes. Absorbance of all samples at 490 nm were taken at zero time (t = 0), measurement of absorbance was continued, until the colour of the β-carotene disappeared in the control reaction (t = 1 h), at 5 min intervals. A mixture prepared as above, without β-carotene, served as blank. All determinations were performed in triplicate.

β-carotene bleaching inhibition was measured by the following formula: (β-carotene absorbance after 1 h/initial absorbance) × 100 [19].

### Determination of antibacterial activity

#### Disc diffusion assay

The disc-diffusion assay [20] was used to determine the growth inhibition of bacteria by the plant essential oil. The bacteria used in this study including *s.a ATCC 25923*, *B.s 166, E. coli GM 109*, *P.ae, S.e ATCC 502* and *L. monocytogynes* were obtained from the laboratory of microbiology, Pasteur Institute, Tunisia. Base plates were prepared by pouring Muller-Hinton (MH) agar into sterile Petri dishes and allowed to set. Plant essential oil aliquots of 10 μl were applied per filter paper disc (Whatman No 6 mm diameter). The disc were air-dried and placed onto the seeded top layer of the agar plates.

Each essential oil was tested (1 disc/plate), with a Penicillin (30 μg/μl), Chloramphenicol (30 μg/μl), and Streptomycin (10 μg/μl) discs as references or positive controls. Methanol saturated discs were used as negative controls. The plates were evaluated after incubation at 37°C for18 h.

Antibacterial activity was expressed as the ratio of the inhibition zone (mm) produced by the plant essential oil and methanolic extract and inhibition zone caused by the reference [21]. The experiments were run in triplicate and averaged.

#### Minimum inhibitory concentration (MIC) of plant extracts and essential oils

*Thymus hirtus sp. algeriensis* essential oils with antibacterial activity were serially diluted to a working concentration ranging from 50–0.5 mg mL^−1^ for determination of their lowest inhibitory concentration (MIC) according to a previously described procedure [22].

## Results and discussion

The amount of total phenolics varied in different *Thymus hirtus sp.algeriensis* methanol extracts and ranged from the lowest value of 7.08 detected for MT samples to the highest level of 8.81 mg GAE/g attributed to MOK collection site (Table 2). Thyme aerial parts methanol extracts should to be rich in flavonoids with amounts varying from 1.08 to 2.25 mg/g RE (Table 2). It should be mentioned that an increase of the phenolic metabolism in these plants may be related to the hard climate conditions (hot temperature, height solar exposure, dryness, short growing season).

**Table 2:** **Major of phenolic compounds (% of total) identified in *Thymus algeriensis *** **methanolic extract by HPLC**

**Compounds**	**Content (μg/g of dry plant material weight)**			**Approximate RT (min)**
	**MT**^**a**^	**MG**^**a**^	**MOK**^**a**^	
phenolic acids				
Hydroxyphenyl acetic acid	914.26 ± 3.42	2053.42 ± 532.2	nd^b^	2.425
gallic acid	723.19 ± 4.1	744.72 ± 12.1	2780.57 ± 492.1	6.0
syringic acid	119.31 ± 4.2	148.45 ± 33.3	-	14.35
Ferulic acid	250.18 ± 3.2	41.64 ± 6.2	4657.94 ± 840.1	24.66
Methyl galate	Nd	229.84 ± 99.2	nd	3.61
Vanillic acid	1189.39 ± 973.3	614.72 ± 41.2	nd	15.96
Hydroxybis	132.2 ± 0.3	5581.77 ± 531.7	nd	3.69
flavonoids				
Tyrosin	5013.06 ± 934.1	59.48 ± 3.9	nd	5.85
flavone	128.6 ± 0.4	65.65 ± 9.6	5512.01 ± 372.2	9.16
Vanillin	1079.26 ± 57.1	126.08 ± 15.8	nd	17.56
(+)- Catechin hydrate	18.01 ± 0.22	4.9 ± 0.7	nd	19.68
Rutin	609.62 ± 0.6	88.54 ± 2.8	nd	23.65

^a^Plant codes are indicated in Table 1; ^b^nd: not detected. Values are means ± SD of three independent replicates from two different samples of each collection sites (n = 6).

**Figure 1:**
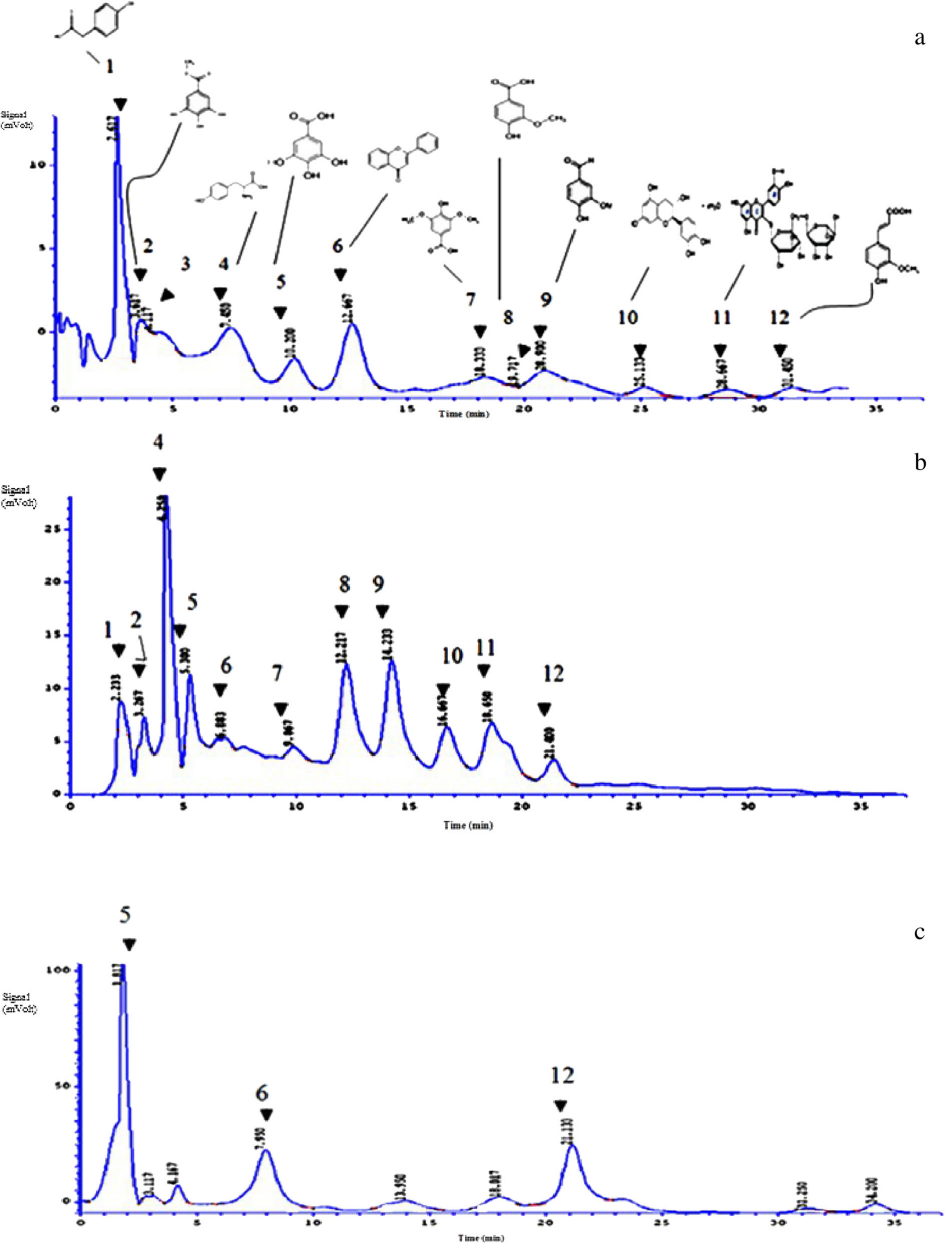
**HPLC chromatogram of methanolic extract of *T.algeriensis *collected from Kairouan (a), Tamerza (b) and Gafsa (c) with response at 280 nm.** Peaks:1, hydroxyphenyl acetic acid; 2, methyl galate; 3, hydroxybis; 4, tyrosin; 5, gallic acid; 6, flavones; 7, syringic acid; 8, vanillic acid; 9, vanillin; 10, (+)- Catechin hydrate; 11, rutin; 12, ferulic acid.

The HPLC chromatographic analysis permitted the identification of eleven phenolic compounds in the methanolic extracts of *Thymus hirtus sp. algeriensis*, including eight phenolic acids ( caffeic acid, ferulic acid, gallic acid, hydroxyphenylic acid, vanillic acid, syringic acid, methyl galate, hydroxybis), and five flavonoids (tyrosin, rutin, (+)- Catechin hydrate, vanillin, flavone). The results are shown in Table 2 and Figure 1. As can be seen data was obtained based on retention comparisons with phenolic acids and other polyphenols standards.

Among the mentioned phenolic compounds, hydroxybis was present in the largest amounts of 5581.77 μg/g followed by flavones and tyrosin. Much lower content was collected for ferulic acid. Whereas the lowest rate was obtained for (+) - Catechin hydrate (4.9 μg/g). It is well-known that gallic acid is one of the major phenolics present in plants. Phenolic acids are usually implicated as natural antioxidants in fruits, vegetables and other plants. For example caffeic acid, ferulic acid, and vanillic acid are distributed in plants (Kingdom) [23]. Differences among phenolic compounds levels can be related to environmental conditions.

However, different results were obtained by Rachid [24] who reported the presence of five 8-c-benzylated flavonoids in *Thymus hirtus sp. algeriensis* extracts, 3 common flavones (apigenin, luteolin, diosmetin); 3 derived from these flavones and two derived from quercetine and kaempferol substituted by p-hydroxybenzyl.

The results obtained by GC/MS analysis of the essential oils are summarized in Table 3 where the compounds are listed in order of their retention index on the HP-5MS column. Determination of the percentage composition of the samples was based on peak area normalization. It is depicted in this table that the chemical composition of Tunisian thyme extracts is characterised by its richness and its variety. Indeed, the number of products in the essential oils is 25 components and the composition of *Thymus* essential oils from three different localities, amounting to a total percentage of 77.75, 77.16, and 92.01%, in Gafsa, Kairouan and Tamerza, respectively.

**Table 3:** **Essential oil composition of aerial parts of *Thymus algeriensis*, cultivated in different locations**

**NO.**	**RI**^**a**^	**Components**^**b**^	**Peak area (%)**			**Identification methods**^**d**^
			**MG**^**c**^	**MT**^**c**^	**MOK**^**c**^	
1	939	α-pinene	-	2.55	3.74	GC-MS
2	946	Camphene	-	1.49	1.83	GC-MS
3	975	Sabinene	-	1.56	-	GC-MS-RT^e^
4	979	β-pinene	-	1.44	1.14	GC-MS
5	1017	α –terpinene	1.18	1.69	-	GC-MS-RI
6	1025	ρ-cymene	3.22	12.43	-	GC-MS-RI
7	1029	Limonene	-	-	0.78	GC-MS
8	1031	1,8-cineole	3.45	14.12	19.96	GC-MS
9	1060	γ-terpinene	2.43	4.30	-	GC-MS
10	1070	*trans*-sabinene hydrate	-	1.21	-	GC-MS-CAS^f^#
11	1089	Terpinolene	1.35	1.25	-	GC-MS
12	1097	*Cis*-sabinene-hydrate	-	1.15	-	GC-MS-CAS#
13	1098	Linalool	18.05	-	-	GC-MS
14	1146	Camphor	13.03	2.16	19.20	GC-MS
15	1165	Pinocarvone	-	-	0.97	GC-MS
16	1169	Borneol	-	2.78	7.64	GC-MS
17	1176	4-carvomenthenol	11.2	-	3.72	GC-MS
18	1177	Terpinen-4-ol	-	33.34	-	GC-MS
19	1205	Verbenone	-	-	1.06	GC-MS
20	1289	Bornyl acetate	5.41	8.34	11.67	GC-MS
21	1420	β-caryophyllene	-	-	0.85	GC-MS
22	1511	γ-cadinene	1.21	-	-	GC-MS
23	1578	Spathulenol	2.80	-	-	GC-MS
24	1582	Caryophyllene oxide	2.09	2.20	4.60	GC-MS
25	1590	Viridiflorol	11.71	-	-	GC-MS
Chemical classes						
		Monoterpene hydrocarbons	8.18	26.71	8.46	
		Oxygenated monoterpenes	51.14	29.76	59.53	
		Sesquiterpene hydrocarbons	1.21	-	0.85	
		Oxygenated sesquiterpenes	16.6	35.54	8.32	
		Total identified, %	77.75	92.01	77.16	
		Essential Oil Yield (%)	2.36	10.82	5.75	

^a^RI (retention index) measured relative to n-alkanes (C10 –C15); ^b^Components listed in order of their retention index on HP on HP-5 column, ^c^Plant codes; ^d^GC-MS: identification based on a high match of mass spectra; ^e^RT: retention time compared with those reported in the literature [25]; ^f^CAS # = Chemical Abstracts service reference number compared with those reported in the literature [26].

The oil yield of *Thymus hirtus sp. algeriensis* collected in Tamerza (Tozeur) is 10.82% (v/w); the main components are terpinen-4-ol (33.34%) and 1.8-cineole (14.12%). Linalool (18.05%) and camphor (13.03%) are the most abundant component in *Thymus hirtus sp. algeriensis* oil collected in Gafsa.

The oil yield of *Thymus hirtus sp. algeriensis* collected in Kairouan is 5.75% and the major components are 1.8-cineole (19.96%) and camphor (19.20%). A similar result was previously described by ElHadj Ahmed [27] who have found that the chemical composition of *Thymus hirtus sp. algeriensis* resulted in the identification of 25 compounds with a dominance of oxygen-containing monoterpenes, the main constituents were linalool (17.62%) and camphor (13.82%), whereas Ben El Hadj Ali [28] showed that camphor characterized few populations from the semi-arid zone of Tunisia.

The essential oil was characterized by very high percentage of monoterpenes and especially the oxygenated ones (29.76-59.53%), which constituted the predominant class as was previously found for *Thymus hirtus sp. algeriensis*[7].

The monoterpene fractions presented the lowest level for the collection of Gafsa which is located in the semi-arid locality, increased for the samples collected in the semi-arid locality Kairouan and reached the highest percentages in the arid sites, located in high altitudes, Tamerza.

The sesquiterpenes were also represented mainly by oxygenated sesquiterpenes (8.32% and 35.54%) in contrast to what has been observed by Ben El Hadj Ali [28], where the amount of oxygenated sesquiterpenes did not exceed 4.6% of the total essential oil of *Thymus hirtus sp. algeriensis*. in fact; samples of Gafsa and Kairouan showed the lowest proportions (8.32% and 35.54%), respectively. MT is remarkable because it includes oils containing the terpenic oxide like 1,8-cineole, and monoterpenic alcohol such as terpinen-4-ol and borneol. The analyzed data illustrated significant differences in the amount of some compounds such as *ρ*-cymene (3.22-12.43%), 1, 8-cineole (3.45-19.96%), linalool (18.05%), terpinen-4-ol (33.34%), camphor (2.16-19.20%), and bornyl acetate (5.41-11.67%) in thyme harvested in different regions.

Thus, the *Thymus hirtus sp. algeriensis* oil analysis revealed that the major components were *ρ*-cymene, 1,8-cineole, linalool, terpinen-4-ol, bornyl acetate and camphor (a bornane derivative). Some components are currently encountered in all species whatever at variable amounts. It concerns monoterpenics phenols (thymol and carvacrol), its monoterpenics hydrocarbures (ρ-cymene and γ-terpinene), oxygenated monoterpenes (borneol and linalool), terpinen-4-ol and 1, 8-cineole [29]. In agreement with our findings, Fatiha [30] obtained a similar result, except for some components like camphor (27.7%) and α-pinene (20.5%). The essential oil of *Thymus hirtus sp. algeriensis* collected from Khedara and Fatoum Souda (Algeria) had the same major components as our samples, but Algerian *Thymus* oils are richer in α-pinene (27.14-25.52%) than in camphor (8.77-8.45%), also in sabinene (5.25-5.61%) and in β-pinene (2.66-3.12%) [31].

Thymol was not detected in all regions described in this research and it was reported at low amounts (<1%) in populations from the same bioclimatic zone described by Zouari [11].

Biogenetic precursor of the phenols were present in a 3.22- 12.43% range ρ-cimene and 2.43-4.3% γ-terpinene. As can be seen in Table 3, Viridiflorol was detected with high rate (11.71%) in a population from the inferior arid zone (MG). A similar result was previously described by Ben El Hadj Ali [28].

Terpinen-4-ol was absent in MG and MOK, except for MT which was distinguished by a high proportion of this constituent (33.34%).

Many factors can be responsible of the variability of thyme extracts chemical composition. The most important are the climate, the soil, the harvest period and the method of preservation and extraction [32-35]. Genetic factors [36] and vegetative cycle [11] can also influence this variability.

In the radical form, DPPH had an absorbance at 517 nm which disappeared after acceptance of an electron or hydrogen radical from an antioxidant compound to become a stable diamagnetic molecule [37].

The method is used to evaluate the antioxidant properties of the thyme methanol extracts and essential oils in comparison with those of known natural and synthetic antioxidants, BHT.

The BHT showed the highest radical scavenging activity, while the thyme methanolic extracts and essential oils demonstrated much lower activity. Table 4 shows the percentage inhibition of free radicals by thyme extracts due to hydrogen donation from the antioxidants. The order of antioxidant activity of thyme extract was: BHT > MT > MG > MOK. The essential oils have an activity in the order: BHT > MT > MOK > MG.

**Table 4:** **Comparison of the content of phenolic and flavonoid and antioxidant activity of *T.algeriensis *** **extracts**^**a **^**suitable for medical uses**

**Sample**	^**b**^**TPC (mg GAE**^**c**^**/g dw**^**d**^**)**	^**e**^**TF (mg RE**^**f**^**/g dw)**	**DPPH Inhibition %**		**ABTS Inhibition %**		**β-Carotene bleaching inhibition %**	
			**M.E**^**g**^	**E.O**^**h**^	**M.E**	**E.O**	**M.E**	**E.O**
MT	7.08 ± 0.7	1.08 ± 0.8	93 ± 0.06	85 ± 0.57	75 ± 0.72	16 ± 0.12	31 ± 0.91	10 ± 0.52
MG	8.7 ± 0.59	1.95 ± 0.4	84 ± 0.034	82 ± 0.52	50 ± 0.96	8 ± 0.7	25 ± 0.08	4 ± 0.44
MOK	8.81 ± 0.12	2.25 ± 0.43	81 ± 0.26	83 ± 0.1	22 ± 0.9	19 ± 0.33	50 ± 0.12	5 ± 0.71
BHT			93 ± 0.25	86 ± 0.62	86 ± 0.37	19 ± 0.81	36 ± 0.66	36 ± 0.66

^a^Values are the mean ± standard deviation (n = 3); ^b^TPC:Total Phenolic Compounds; ^c^GAE. Gallic acid equivalents; ^d^Dry weight basis of the original sample of plant parts; ^e^TF: Total Flavonoids; ^f^RE. Rutin equivalents; ^g^Methanolic extracts; ^h^Essential oils.

These results concur with other researchers who have also reported that another species of thymus like *Thymus satureidoїdes* had the best antioxidants activities (74.50%), nevertheless inferior to those of BHT (98.59%), and the same thing for *T. vulgaris* with an antioxidant activity of about 57.46% [38]. In disagreement with our result. Gianni [39] found that the percentage inhibition of *T.vulgaris* (90.9 ± 0.64%) is superior to those of BHA (68.74 ± 0.61%). Essential oil showed the lowest scavenging effect on DPPH. Our results are in accordance with other investigators [40] who have also reported that the *Thymus capitatus* oil had a weak antioxidant activities, essential oil concentrations are 12.0; 15.0; 25 and 62.5 μg/ml which had the scavenging effect on DPPH, respectively, 27 ± 2.0, 30 ± 0.7 and 40 ± 0.3%. The MT extract showed the highest radical scavenging activity (RSA) among all tested methanol extracts, followed by MG and MOK. In contrast, MOK showed the lowest RSA values (80%) at 50 μg/ml.

**Figure 2:**
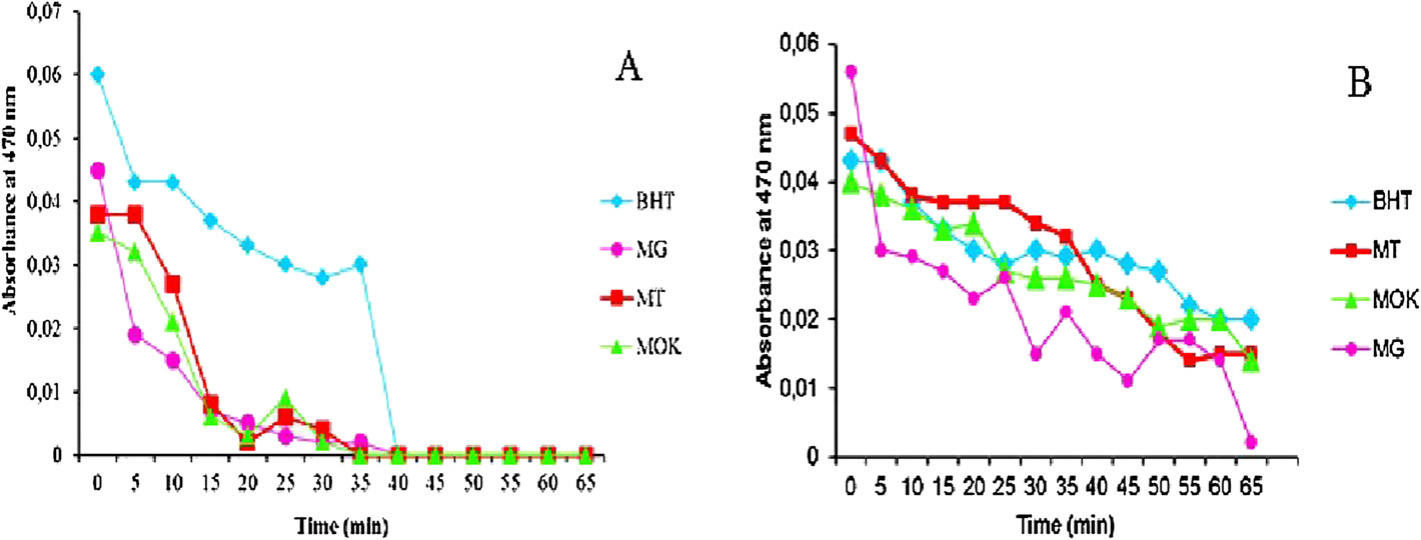
The antioxidant activity of essential oils (A) and methanolic extracts (B) of thymus hirtus sp. algeriensis using the β-carotene bleaching, BHT (◆), MG (●), MOK (▲), MT (■).

Although the DPPH radical scavenging ability of the extracts and essential oils were significantly lower than those of BHT, it was evident that the extracts did show the proton-donating ability and could serve as free radical inhibitors or scavengers, acting as possibly as primary antioxidants.In the case of β-carotene /linoleic acid test results, the presence of thyme extract can hinder the extent of β-carotene bleaching by acting on the linoleate-free radical and other free radicals formed in the system. Accordingly, the absorbance decreased rapidly in samples without antioxidant whereas, in the presence of MT methanolic extract, they retained their color and thus absorbance, for a longer time. Figure 2 shows the absorbance of the total thyme methanolic extracts and essential oils with the BHT. The antioxidant power decreased in the order BHT > MT > MOK > MG for methanol extracts and BHT > MOK > MG > MT for essential oils. The decreasing of absorbance of β-carotene with or without extract (or essential oils) is measured with time. The extract (or essential oils) avoid the bleaching of β-carotene with comparison with the control. The absorbance of the control demonstrate a decreasing after 15 min. Aerial part oils had the weak antioxidant activities, and its inhibition were of about third of that of BHT, in contrast to the methanolic extracts of the same plants, which presented activities comparable of that of BHT. But for essential oil of MT the rate is slow to maintain the absorbance at 0.04 after 35 min.

The double bonds of β-carotene and saturated fatwere attaqued by radicals. Results clearly demonstrated that extracts and essential oils of *Thymus hirtus sp. algeriensis* delayed the degradation of β-carotene.

Proton radical scavenging is an important attribute of antioxidants ABTS, a protonated radical, has characteristic absorbance maxima at 743 nm which decreases with scavenging of the proton radicals [41]. The antioxidant activity of thyme extracts from methanol was evaluated by means of ABTS assay, and the results are shown in Table 4. Apparently, the antioxidant activity of thyme extracts was found to be in the order: MT > MOK > MG. Of the three samples tested, MT had the highest ABTS values. The results obtained suggest that the higher the polarity of thyme extracts, the stronger is the antioxidant activity.

To establish the relationship between phenolic compounds and the antioxidant capacity, linear correlation coefficients were calculated and results are shown in Table 5. Positive significant (*p* < 0.05) correlations confirmed between vanillic acid, tyrosin, vanillin, (+)- Catechin hydrate, rutin and the DPPH assay proved the significance of these compounds in the scavenging power of *Thymus hirtus sp. algeriensis* extracts. Similarly, vanillic acid showed a positive significant (*p* < 0.05) correlation with the ABTS scavenging activity. On the contrary, total phenolic and total flavonoid contents illustrated negative significant (*p* < 0.05) correlations with the DPPH test. Previous investigations on several Lamiaceae species such as *Rosmarinus officinalis*[42], *Thymus vulgaris*[43], *Marrubium globosum*[44], *Salvia officinalis*, *S. verbenaca*, *S. aegyptiaca* and *S. argentea*[45] demonstrated close correlations between polyphenolics and antioxidant activity, which could support the effectiveness of these compounds as free radical-scavengers and antioxidants. The good relationship between the less represented phenolic compound namely (+) - Catechin hydrate and the DPPH assay (Table 5) may support the contribution of minor compounds in synergy with major compounds to the resulting antioxidant activity.

**Table 5:** Correlation coefficients between phenolic contents versus the antioxidant capacities determined by DPPH, ABTS and β-carotene bleaching

	**DPPH**	**ABTS**	**β-carotene bleaching**
Hydroxyphenyl acetic acid	0.200	0.470	−0.940
Gallic acid	−0.700	−0.890	0.970
Syringic acid	0.500	0.780	−1.000
Ferulic acid	−0.700	−0.860	0.980
Methyl galate	−0.300	0.030	−0.690
Vanillic acid	1.000	1.000	−0.740
Hydroxybis	−0.300	0.050	−0.700
Tyrosin	1.000	0.850	−0.300
Flavone	−0.700	−0.880	0.980
Vanillin	1.000	0.900	−0.390
(+)- Catechin hydrate	1.000	0.960	−0.530
Rutin	1.000	0.910	−0.410
Total phenolic content	−1.000	−0.880	0.340
Total flavonoid content	−1.000	−0.950	0.520

The antibacterial activity of essential oil was tested against six different genera of bacteria namely *E.coli*, *P. ae*, *S. e*, *S. a*, *B. s* and *L. monocytogynes*. The essential oil from *Thymus hirtus sp. algeriensis* of 3 regions in Tunisia has a considerable inhibitory effect on all tested bacteria; it demonstrated different degrees of growth inhibition. Results of antibacterial activity of *Thymus hirtus sp. algeriensis* oils are summarized in Table 6. Results clearly demonstrated that MOK oil showed the lowest antibacterial activity among the tested oils; with i.z. (inhibition zone) 9.0-36.0 mm; MT and MG possessed almost the same activity with i.z.30.0-74.0 mm. Penicillin showed activity with i.z.0-10 mm for *L. monocytogynes*. The antibacterial activity of *Thymus hirtus sp. algeriensis* oils tested was found to have an effect on the gram-positive bacteria *S. a ATCC 52923* and *B. s 106* which seemed to be more easily inhibited than the Gram-negative bacteria, namely *E. coli GM 109*, *P. ae* and *S. e ATCC 502*. An important characteristic of this finding totally agrees with the observation derived from studies with essential oils from other thyme species [46].

**Table 6:** **Antibacterial activity of the essential oil of *Thymus algeriensis***

**Micoorganisms**	**Essential oil**						**Antibiotics**					
	**MOK**		**MT**		**MG**		**SM**^**c **^**(10 μg/μl)**		**CP**^**d **^**(30 μg/μl)**		**PC**^**e **^**(30 μg/μl)**	
	**DD**^**a**^	**MIC**^**b**^	**DD**	**MIC**	**DD**	**MIC**	**DD**	**MIC**	**DD**	**MIC**	**DD**	**MIC**
*Staphylococcus aureus ATCC 25923*	22	4.50	63	1.50	63	1.70	15	20.00	33	2.00	nt	nt
*Bacillus subtilis 166*	36	5.50	30	4.00	25	4.50	22	5.00	20	1.00	nt	nt
*Salmonella enteridis ATCC 502*	9	22.00	43	2.00	65	1.50	13	20.00	27	1.50	nt	nt
*E.coli GM 109*	30	4.00	50	1.80	30	4.20	15	23.00	27	1.50	nt	nt
*Pseudomonas aeruginosa*	9	22.00	74	0.90	30	4.50	17	20.00	28	1.50	nt	nt
*Listeria monocytogynes*	20	7.50	45	2.00	32	4.00	nt^f^	nt	nt	nt	10	22.00

^a^DD, disc diffusion method as recommended by NCCLS. Diameter of zone of inhibition (mm) including disk diamater of 6 mm, ^b^MIC, minimum inhibitory concentration; Value given as mg/ml (for the essential oil) and as μg/ml (for antibiotics); ^c^SM, Chloramphenicol (30 μg/μl); ^d^CP, Streptomycin (10 μg/μl); ^e^PC, Penicillin (30 μg/μl); ^f^nt, not tested.

Soković [47] reported the effect of essential oil of *T. vulgaris* on Gram (+) bacteria as *S.a*, *B.s*, with same i.z (28 mm), instead, Bektas [48] reported that *T. hyemalis*, essential oil showed weaker antimicrobial activity, he found that in the presence of this sample, no activity was observed against *P.ae* and *L.monocytogynes*, but a great effect on Gram (+) bacteria as *B.s*, *B.c* and *S.a* with i.z, respectively, 17.30 ± 0.64, 20.50 ± 0.46 and 15.00 ± 0.74 mm. It is obvious that Gram-negative bacteria were found to be sensitive to *Thymus hirtus sp. algeriensis* oils, despite that they have an outer layer surrounding their cell wall that act as permeability barrier, limiting the access of hydrophobic compounds [49]. MT was more effective in inhibiting all tested bacteria, than those of MG and MOK. The microorganism *E.coli*, which is already known to be multi-resistant to drugs, was susceptible to different antibiotics, had its growth inhibited by essential oil of *Thymus hirtus sp. algeriensis* (37–63 mm), this was due to the loss of resistance, probably because of the loss of plasmids. Our results are comparable to those of Giweli [50] who found that the oil of a Mediterranean species *satureja thymbra* (family Lamiacea) showed bacteriostatic activity at 0.001-0.1 mg/mL and was bactericidal at 0.002-0.2 mg/mL; fungistatic effects at 0.001-0.025 mg/mL and fungicidal effects at 0.001-0.1 mg/mL. From our results it can be seen that MIC_s_ are generally lower for the essential oil investigated in this research. *Thymus hirtus sp. algeriensis* essential oil showed slightly higher antibacterial activity than Chloramphenicol, Ampicillin and Streptomycin.

Essential oil of thyme can have preventive effect by the means of antioxidants properties of its components. It seems evident that there is a relationship between the high activity of *Thymus hirtus sp. algeriensis* oils and the presence of monoterpene alcohol, such as terpinen-4-ol and linalool.

Earlier reports had claimed the effect of cyclic monoterpenes. This observation was consistent with a previous report, where 1, 8-cineole and borneol were found to have a moderate antibacterial activity [51]. In disagreement with this finding, previous results showed that greater antimicrobial potential could be ascribed to the oxygenated terpenes, especially phenolic compounds. The antibacterial property of mostly essential oils is suspected to be associated with the high percentage of caryophyllene oxide, α-pinene, and β-pinene, and 1,8-cineole, which are known to possess strong antibacterial activity [52]. Andrews [53] found that α-pinene (2 mM) disrupted the cytoplasmic membranes of *Saccharomyces cerevisiae* and the Gram-positive organism *Bacillus thuringiensis*, but that Gram-negative bacteria were more resistant to terpenes. β-pinene inhibited respiration at the cytochrome b portion of the electron transport chain of yeast cells [54].

It appears that the small molecular weight of essential oil compounds allows them to penetrate the inner membrane of Gram-negative bacteria [55]. It can be seen that the antibacterial activity of MG oil responded to great percentage of linalool, camphor, terpinen-4-ol and Viridiflorol. These results concur with other researchers who have also reported that linalool has a strong effect against a number of different bacteria [56]. In disagreement a previous result corroborated that the essential oil from *Thymus zygis* containing 39% linalool had no bacteriostatic activity against isolates of *E. coli* unless thymol or carvacrol was also present. The oil of MOK exhibited moderate activity against *S. a ATCC 25923* and *L. monocytogynes* (MIC_s_, 4.5 to 7.5 mg/ml) and slightly lower antibacterial effect were observed against *P. ae* and *S. e ATCC 502* (22.00 mg/ml).

It is extremely important to point out that, antibacterial effects of essential oils in *Thymus hirtus sp. algeriensis* are strictly related with the presence of 1.8-cineole which is present in great proportion in MT (14.12%) and MOK (19.96%).

The above screening result enumerates the existing potential of plant essential oil to be used as suitable constituent in pharmaceuticals and food preservatives of plant origin for treating plant and animal diseases caused by pathogens and food spoiling microorganisms.

## Conclusion

The beneficial effects of flavonoids in cancer therapy have often been linked to their ability to act as antioxidants, which includes their reducing capacities and ROS-scavenging capabilities.

## Abbreviations

HPLC: Height pressure liquid chromatography; DPPH: 2, 2-diphenyl-1-picrylhydrazyl; ABTS: 2, 2-Azinobis-3-ethylbenzothiazoline-6-sulfonate; BHT: Butylatedhydroxytoluene; FC: Folin-Ciocalteu; MoO^4+^: Molybdenum oxide; GAE: Gallic acid equivalents; Na_2_CO_3_: Sodium bicarbonate; RE: Rutin equivalents; Abs: Absorbance; K_2_S_2_O_8_: Potassium persulfate; BHA: Butylatedhydroxyanisol; RSA: Radical scavenging activity; *s.a*: *staphylococcus aureus*; *B.s*: *Bacillus subtilis*; *E. coli*: *Escherichia coli*; *P.ae*: *Pseudomonas aeruginosa*; *S.e*: *Salmonella enteredis*; *L. monocytogynes*: *Listeria monocytogynes.*

## Competing interests

The authors declare that they have no competing interests.

## Authors’ contributions

GF, BFM participated in the design of the study and performed the statistical analysis. GF, BFM and MM have been involved in drafting the manuscript and revising it critically for important intellectual content, LA have given final approval of the version to be published. All authors read and approved the final manuscript.
